# Screening for acute hepatic porphyria in postural tachycardia syndrome

**DOI:** 10.1007/s10286-025-01153-5

**Published:** 2025-08-26

**Authors:** Naome Mwesigwa, Hadley Williamson, Shalonda Turner, Mehr E. Pouya, Tan Ding, Ortiz J. Pedro, Karl E. Anderson, Cyndya A. Shibao

**Affiliations:** 1 Division of Clinical Pharmacology, Department of Medicine, Vanderbilt University Medical Center, 506 Robinson Research Building, Nashville, TN 37232-880 USA; 2 Galveston Porphyria Laboratory, Division of Gastroenterology and Hepatology, Department of Internal Medicine, University of Texas Medical Branch, Galveston, TX USA

**Keywords:** Postural tachycardia syndrome (POTs), Acute hepatic porphyria, Urinary porphobilinogen, Delta-aminolevulinic acid

## Abstract

**Purpose:**

Postural orthostatic tachycardia syndrome (POTS) is characterized by an excessive heart rate increase upon standing, often associated with dizziness, gastrointestinal symptoms, and decreased functional capacity. Acute hepatic porphyrias (AHP) are rare metabolic disorders with nonspecific neurovisceral and autonomic symptoms, some of which overlap with POTS. The purpose of this study was to evaluate AHP by molecular and biochemical testing in patients with POTS.

**Methods:**

We studied 50 patients diagnosed with POTS and gastrointestinal symptoms at the Vanderbilt Autonomic Dysfunction Center. They underwent neuro-hormonal evaluation for POTS and genetic and biochemical screening for AHP. Genetic testing was aimed mainly at the four genes relevant to AHPs. Porphobilinogen (PBG), delta-aminolevulinic acid (ALA), and total porphyrins were measured in urine with normalization to creatinine.

**Results:**

The average age of the patients was 33 ± 8.6 years, 96% were female, and the average BMI was 28 ± 7.2 kg/m^2^, average systolic blood pressure was 120 ± 15.5 mmHg, average heart rate was 77 ± 13.6 bpm at baseline, and average SBP was 126 ± 19.1 mmHg. A heart rate of 111 ± 15.8 bpm at 10 min upright, showed normal cardiovascular reflexes. The COMPASS-31 total score was 32 ± 8.4, with a normal autonomic function test. Urine PBG averaged 1 ± 0.7 mg/g creatinine, ALA 2 ± 0.9 mg/g creatinine, and total porphyrins 172 ± 74.2 mmol/g creatinine, which were all normal. None had variants in the four genes associated with AHPs. Three patients were heterozygous for a common low expression ferrochelatase gene variant (FECH).

**Conclusions:**

We found no evidence of AHP in patients with POTS with uncontrolled gastrointestinal symptoms, suggesting that screening for AHP, a rare genetic disorder, may not be warranted.

## Introduction

Postural tachycardia syndrome (POTS) is characterized by an excessive increase in heart rate upon standing, often accompanied by orthostatic intolerance, fatigue, and a wide range of neurological symptoms [1]. Prior to the COVID-19 pandemic, it was estimated to affect 1–3 million individuals in the USA, of whom 80–85% are women [2]. Approximately half of patients with POTS have excessive sympathetic (SNS) activity as measured by elevated upright plasma norepinephrine (NE) levels, which contributes to associated cardiovascular symptoms such as lightheadedness, tachycardia, palpitations, and blurred vision [3]. POTS is also associated with gastrointestinal symptoms, including abdominal pain, nausea, vomiting, bloating, early satiety, and constipation, as well as numbness and tingling in the lower extremities [4, 5]. These symptoms may be intermittent or constant and possibly exacerbated by other illnesses and infections. The causes of POTS are not fully known and may not be the same in all patients.

Acute hepatic porphyrias (AHP) are a group of four rare, inherited disorders, each due to an inherited deficiency of four of the eight enzymes in the heme biosynthetic pathway [6, 7]. Intermittent abdominal pain, nausea and vomiting, constipation, abdominal distension and bloating, anorexia, paralytic ileus, as well as tachycardia and other autonomic abnormalities, are common in those who develop symptoms of AHP, but these are intermittent and more common in women [8]. The three most common AHPs are acute intermittent porphyria (AIP), variegate porphyria (VP), and hereditary coproporphyria (HCP), in that order, and are low-penetrance disorders with autosomal dominant inheritance. δ-aminolevulinic acid (ALA) dehydratase porphyria (ADP) is ultra-rare and autosomal recessive [9]. AHP is readily diagnosed by elevations in urine ALA, porphobilinogen (PBG), and porphyrins at the time of symptom onset and often between symptom episodes [10]. Native or altered forms of these intermediates may be neurotoxic. It remains possible, but uncertain, that symptoms may occur due to deficiencies in vital hemoproteins, even in the absence of elevations of ALA, PBG, and porphyrins [11]. Genetic testing to identify pathogenic mutations is increasingly relied upon for detecting latent cases of AHP [12].

Because the presenting symptoms in patients with POTS and abdominal pain overlap with those of AHP, for which specific treatments are available, we sought to determine if AHP was present in a series of these patients. We applied biochemical testing to detect active AHP and genetic testing for pathogenic mutations that can identify AHP during the latent stages of these diseases. We also studied the neuro-hormonal characteristics of this POTS subgroup with recurrent episodes of abdominal pain.

## Methods

### Study population

This prospective study was conducted between 2021 and 2024 at the Vanderbilt Autonomic Dysfunction Clinic (ADC). Vanderbilt Human Research Protection Program approved the protocol, and all subjects provided informed consent. The study was registered in clinicaltrials.gov (NCT05344599). Fifty patients, 18–65 years of age with an established diagnosis of POTS and recurrent abdominal pain, and other symptoms such as nausea, constipation, vomiting, diarrhea, and bloating, were enrolled after signing a consent form for autonomic testing and additional biochemical and genetic testing for AHP. All patients had met the criteria for AHP, which includes having either a family history of acute hepatic porphyria, unexplained recurrent (more than one), prolonged (> 24 h) episode of severe, diffuse (poorly localized) abdominal pain and at least two of the following: red to brownish urine, blistering skin lesions on sun-exposed areas, peripheral nervous system manifestations occurring around the time of abdominal pain (motor neuropathy (paresis), sensory neuropathy (numbness, tingling, limb pain), central nervous system manifestations occurring around the time of abdominal pain (confusion, anxiety, seizures, hallucinations), autonomic nervous system manifestations occurring around the time of abdominal pain (hyponatremia [Na < lower limit of normal]), tachycardia, hypertension, nausea and vomiting, and constipation. In addition, they completed a questionnaire about their past medical history, specifically related to gastrointestinal symptoms. POTS was defined as recurrent pre-syncopal symptoms for more than 6 months and orthostatic tachycardia (> 30 bpm increases in HR within 10 min after assuming an upright position). Autonomic function tests to assess adrenergic and cardiovagal responses performed at Vanderbilt ADC within the prior year included orthostatic blood pressure and heart rate response to tilt, heart rate response to deep breathing, the Valsalva ratio, and beat-to-beat blood pressure measurements during phases II and IV of the Valsalva maneuvers. Participants completed autonomic symptom assessment (COMPASS-31) and quality of life EQ-5D questionnaires [13, 14]. Complete blood count, comprehensive metabolic panel, serum ferritin, iron levels, and plasma catecholamines were obtained within the prior 3 months.

Screening for active AHP, urine PBG, ALA, and total porphyrins were measured in spot urine samples from all 50 participants by the Galveston Porphyria Laboratory at the University of Texas Medical Branch, with results normalized to urine creatinine. Genetic testing was performed by Invitae Inc. (San Francisco, CA) in all 50 patients. However, results were provided for 44 as 6 patients had failed results due to the inability of the specimens to meet quality metrics. This test aimed to detect variants in ten genes associated with the various porphyrias, including the four genes specific to AHP.

### Data analysis

Standard graphing and screening techniques were used to detect outliers and ensure data accuracy. Mean and standard deviation values for continuous endpoints and percentages of categorical factors were calculated. All analyses used SPSS version 23.0 (SPSS, Chicago, IL) and R version 4.3.2 (R Core Team, Vienna, Austria, 2023).

## Results

**Fig. 1:**
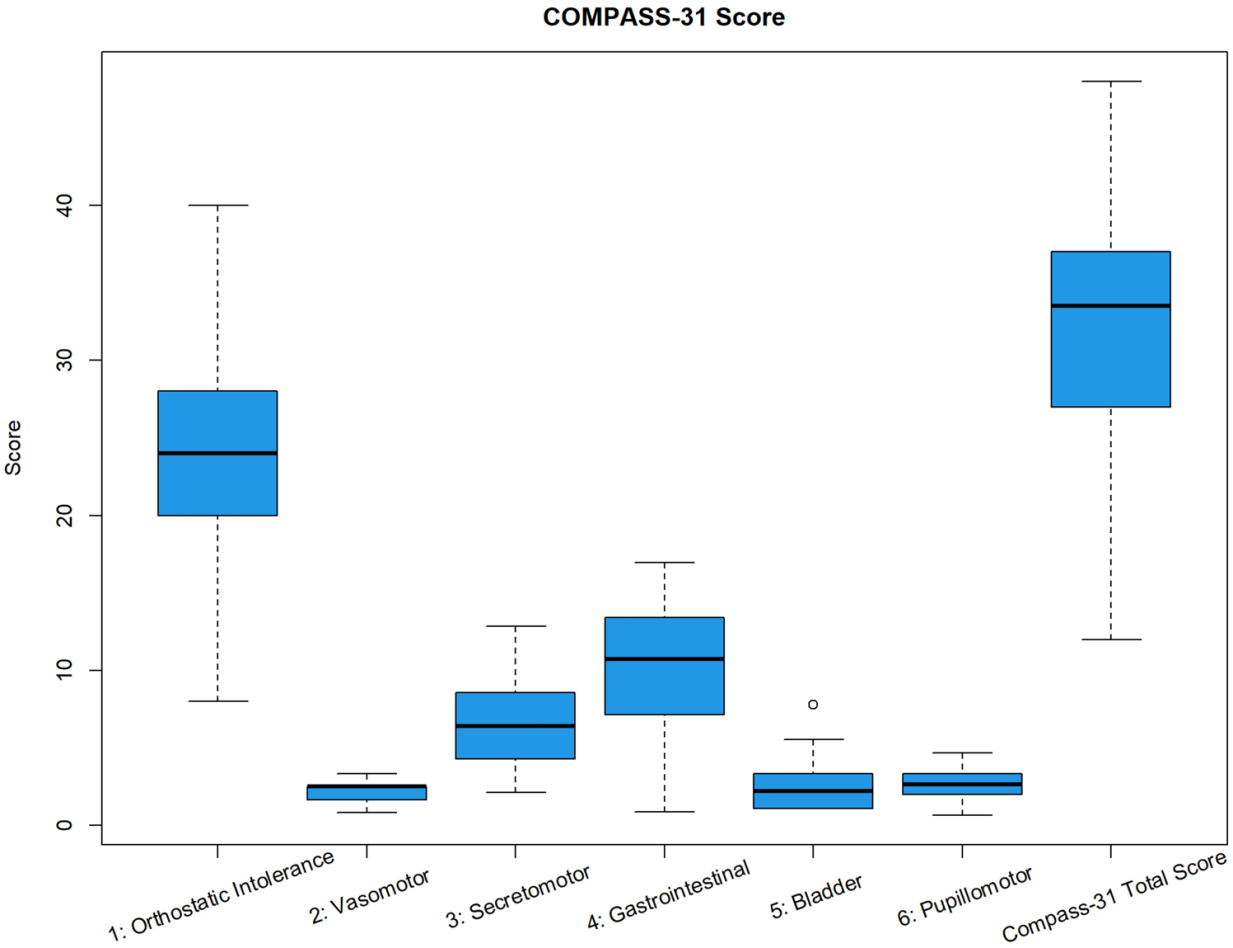
COMPASS-31 subscale scores for different autonomic domains. This figure illustrates the distribution of COMPASS-31 scores across different autonomic domains. Each bar represents the mean score for each domain with standard deviation (SD)

Of 196 patients with POTS pre-screened, 61 fulfilled criteria for AHP screening, of which 50 provided written informed consent and completed the study. These participants were 96% female, aged 33 ± 8.6 years, with a height of 165 ± 8.3 cm, a weight of 78 ± 26.7 kg, and a BMI of 28 ± 7.2 kg/m^2^. Health-related quality of life was assessed using the EQ-5D instrument in all 50 patients, and results demonstrated substantial impairment. Over half reported problems with self-care (52%), the majority reported moderate-to-severe pain/discomfort (78%), and anxiety/depression (44%). The average self-rated health score (EQ-VAS) was 56 ± 17.3, with values ranging from 45 to 73. EQ-VAS ranges from 0 to 100, where higher scores indicate better self-rated health. The autonomic symptom burden was evaluated using the COMPASS-31 questionnaire, and the mean total score was 32 ± 8.4. This scale ranges from 0 to 100, with higher scores reflecting greater symptom burden. Scores of the different domains were orthostatic intolerance scores of 25 ± 6.4, vasomotor 2 ± 0.9, secretomotor 6 ± 2.8, gastrointestinal 10 ± 3, bladder 3 ± 1.8, and pupillomotor 3 ± 0.9 (Fig. 1).

The mean systolic blood pressures and heart rates, supine, were 120 ± 15.5 mmHg and 77 ± 13.6 bpm, respectively, and 126 ± 19.1 mmHg, 111 ± 15.8 bpm after 10 min of standing, with a delta heart rate of 35 ± 12.8 (Table 1). Hematocrit, serum iron levels, electrolytes, and liver function tests were normal (Table 2). The mean seated plasma norepinephrine level was 444 ± 176.4 pg/mL and eGFR was 95 ± 36.5. Notably, 53% of patients reported a history of Ehlers–Danlos syndrome, 22% reported mast cell activation syndrome, and 4 ± 1.5 episodes of abdominal pain per week (Table 3). Urine ALA, PBG, and total porphyrins were normal in all 50 participants. Among the 50 patients, 3 individuals were identified as carriers of AHP, suggesting a prevalence rate of 6% in this cohort, all with one pathogenic variant identified as a FECH gene mutation, associated with autosomal recessive erythropoietic protoporphyria; 76% were negative, 6% were uncertain results, and 12% failed results.

**Table 1:** Orthostatic measurements: blood pressure and heart rate responses

Hemodynamic parameters	*n* = 50	Baseline (mean ± SD)	Standing 1 min (mean ± SD)	Standing 3 min (mean ± SD)
SBP (mmHg)		120 ± 15.5	124 ± 16.8	124 ± 15.8
DBP (mmHg)		75 ± 10.8	81 ± 10.7	83 ± 11.5
HR (bpm)		77 ± 13.6	99 ± 21.3	104 ± 20.6

This table presents the orthostatic measurements of blood pressure and heart rate. The table includes baseline measurements and values recorded 1, 3, 5, and 10 min after standing. Data are presented as means with standard deviations (SD)

**Table 2:** Descriptive statistics of hematologic and biochemical parameters in study participants

Test	Mean ± SD	Median (min, max)
Hemoglobin (g/dL)	14 ± 1.3	14 (10, 16)
Mean corpuscular volume (Fl)	87 ± 14.7	90 (11, 100)
Serum iron (μg/dL)	96 ± 43.7	92 (29, 231)
Serum ferritin (ng/mL)	63 ± 70.3	37 (6, 313)
Chloride (mmol/L)	106 ± 2.5	106 (100, 112)
Potassium (mmol/L)	4 ± 0.5	4 (4, 6)
Sodium (mmol/L)	140 ± 1.9	140 (135, 144)
Blood urea nitrogen (mg/dL)	11 ± 3.4	11 (0.4, 19)
Creatinine (mg/dL)	1 ± 0.1	1 (0.6, 1.1)
Glucose (mg/dL)	82 ± 14.3	80 (51, 121)
Albumin (g/dL)	5 ± 0.5	5 (3.7, 7.4)
Total bilirubin (mg/dL)	1 ± 0.6	0.4 (0.2, 4.1)
Alanine aminotransferase (U/L)	37 ± 100.0	16 (6, 721)
Alkaline phosphate (U/L)	72 ± 26.7	67 (14, 158)
Aspartate aminotransferase (U/L)	32 ± 67.0	21 (11, 492)

This table summarizes the mean (standard deviation) and median (minimum, maximum) values for key laboratory measurements, including hemoglobin, mean corpuscular volume, serum iron, serum ferritin, liver function test, renal functional test, and serum electrolytes

**Table 3:** Prevalence of reported symptoms and comorbid conditions among study participants

Symptoms and clinical history	Yes (%)	No (%)
History of mast cell activation syndrome (MCAS)	22%	78%
History of Ehlers–Danlos syndrome	53%	47%
Excessive sweating	62%	38%
Urinary symptoms (incontinence or frequency)	58%	42%
Pain, numbness, or discoloration in feet	90%	10%
Light-headedness or dizziness	98%	2%
Mean abdominal pain episodes per week (mean ± SD)	4 ± 1.5	
*Gastrointestinal complaints*		
Symptoms	Yes (%)	
Bloating	80%	
Constipation	74%	
Nausea	80%	
Diarrhea	66%	

This table presents the proportion of participants reporting various symptoms and medical conditions. Data include histories of mast cell activation syndrome (MCAS) and Ehlers–Danlos syndrome, as well as common autonomic and gastrointestinal symptoms

## Discussion

This study aimed to investigate the frequency of AHP among patients with POTS with visceral symptoms [15]. Our main finding was that there was no evidence of AHP in patients with POTS with gastrointestinal (GI) symptoms, and porphyrin metabolites were not associated with disease burden.

Abdominal pain frequently causes significant morbidity in patients with POTS. The underlying pathophysiology is multifactorial and could involve both autonomic and non-autonomic mechanisms. Frequently, patients present symptoms such as early satiety, constipation, bloating, and gastrointestinal dysmotility, including delayed gastric emptying and intestinal dysmotility. Of note, small intestinal bacterial overgrowth can also be seen in patients with POTS [16]. Mast cell activation syndrome (MCAS) is also a common comorbid condition in POTS, which induces abdominal cramping and bloating associated with enhanced secretion of histamine and inflammatory cytokines [1, 17].

In this study, we primarily enrolled patients with significant GI symptoms as shown by the elevated COMPASS-31 score, particularly the GI symptom scores. Normal COMPASS-31 is typically less than 10 in normal individuals [13]. Unfortunately, there is currently no standardized clinical evaluation protocol for abdominal pain or hypersensitivity in POTS. However, the clinical approach begins with a detailed history and physical exam focused on autonomic symptoms, bowel habits, dietary triggers, and pain characteristics. Stool studies may be conducted in case of diarrhea or suspected malabsorption. Further, if gastroparesis is suspected or another gastrointestinal motility disorder, patients undergo gastric emptying scintigraphy. For suspected MCAS, serum tryptase levels are measured along with 24-h urine testing for *N*-methylhistamine and prostaglandin D2 metabolites [4, 18]. In a large percentage of patients, the underlying cause of abdominal pain is not detected. Therefore, other causes should be considered.

The prevalence of AHP carriers is approximately 0.059% in the general public. It is worth noting that 6% of the patients were carriers of the FECH gene, which was significantly higher than the 1–2% rate in the general population [19]. The ferrochelatase gene (FECH) is found mostly patients with autosomal recessive erythropoietic protoporphyria (EPP). The four genes of particular interest regarding AHPs are hydroxymethylbilane synthase (HMBS), coproporphyrinogen oxidase (CPOX), protoporphyrinogen oxidase (PPOX), and δ-aminolevulinic acid dehydratase (ALAD) [11].

In addition, even when AHP shares some clinical features with POTS, such as abdominal pain, autonomic dysregulation, and neurovisceral symptoms, the results did not support a significant association between AHP and POTS in the cohort. Notably, the biochemical testing revealed no increase in AHP markers such as urinary porphobilinogen or ALA, suggesting that AHP prevalence among patients with POTS is not higher than in the general population.

Abdominal pain in patients with POTS may have multiple etiologies, but screening for AHP, a rare disorder, appears to be unnecessary, and more emphasis should be put on ruling out more likely etiologies, including intestinal dysmotility and gastroparesis. However, this study raises the need to investigate further the additional variants identified in this cohort if found in more patients with POTS.

Of note, the study had several limitations: the diagnosis of MCAS and GI dysmotility relied on passive reporting through questionnaires and surveys without supporting objective data.Inclusion of a data availability statement is preferred for this journal. If applicable, please provide one.The data that supports the findings of this study are available from the corresponding author upon request.

Even though we enriched the cohort by only including patients with POTS with uncontrolled GI symptoms, who met criteria for AHP screening, the sample size was still small.

## Data Availability

The data that supports the findings of this study are available from the corresponding author upon request.
